# Immune Checkpoint Inhibitor–induced Myocarditis: A Case Report of Complete Heart Block and Challenges in a Patient on Pembrolizumab

**DOI:** 10.19102/icrm.2025.16033

**Published:** 2025-03-15

**Authors:** Viraj Panchal, Elina Momin, Shubhika Jain, Anaiya Singh, Guntas Ghuman, Vijaywant Brar

**Affiliations:** 1 Department of Medicine, Smt. NHL Municipal Medical College and SVPISMR, Ahmedabad, Gujarat, India; 2 Department of Internal Medicine, Louisiana State University, Shreveport, Louisiana, USA; 3 Department of Internal Medicine, Government Medical College and Hospital, Chandigarh, India; 4 Department of Cardiology, Louisiana State University, Shreveport, Louisiana, USA

**Keywords:** Case report, complete heart block, myocarditis, pembrolizumab

## Abstract

Immune checkpoint inhibitors (ICIs) have revolutionized cancer treatment by modulating immune responses, leading to enhanced anti-tumor activity. ICIs, including agents targeting cytotoxic T-lymphocyte antigen 4, programmed cell death 1, and programmed cell death ligand, are now widely used in various malignancies, either as monotherapy or in combination with chemotherapy, radiotherapy, or targeted therapies. However, ICIs are associated with immune-related adverse events, affecting multiple organ systems, with myocarditis emerging as a rare but potentially fatal complication. We present a 67-year-old man with a history of prostate and renal cell carcinoma treated with pembrolizumab and lenvatinib, who developed myocarditis secondary to ICI therapy. The patient initially presented with generalized fatigue and bradycardia, later progressing to more severe symptoms, including sinus bradycardia and elevated troponin levels. An electrocardiogram revealed a sinus rhythm with first-degree atrioventricular block, non-specific intraventricular conduction delay, and elevated high-sensitivity troponin levels progressively increasing to 50,000 pg/mL. A comprehensive diagnostic workup ruled out ischemic causes, leading to the diagnosis of ICI-induced myocarditis. The patient was treated with high-dose corticosteroids, intravenous immunoglobulin, and temporary pacemaker insertion, resulting in clinical improvement. This case highlights the need for vigilance and prompt intervention in patients receiving ICI therapy, as early recognition and treatment of myocarditis are crucial to optimizing patient outcomes in this high-risk population.

## Introduction

One of the most exciting developments in cancer treatment is the modulation of immune response using immunotherapy. Among these, immune checkpoint inhibitors (ICIs) are becoming the standard treatment modalities for malignancies, along with the combination of chemotherapy, radiotherapy, and targeted therapy.^1^ These are monoclonal antibodies directed against the host immune-negative checkpoints, such as cytotoxic T-lymphocyte antigen 4 (CTLA-4), programmed cell death ligand (PD-L1), programmed cell death 1 (PD-1), and lymphocyte activating gene 3 (LAG-3), which leads to the activation of T-cell activity, eventually causing activation of the immune system to produce an anti-tumor response.^2–4^ The U.S. Food and Drug Administration has approved seven ICIs, which include ipilimumab (anti–CTLA-4), nivolumab, pembrolizumab, cemiplimab (anti–PD-1), avelumab, atezolizumab, and durvalumab (anti–PD-L1), with expansions in their indication in cancer therapy; some are even being used as first-line therapies.^5^ Despite their anti-tumor efficacy, ICIs demonstrate inherent immune-related adverse events (IRAEs) involving multiple organ systems such as the colon, lung, liver, thyroid, and heart.^6^

The most common fatal IRAE is colitis, with an associated mortality of up to 5%.^7^ The occurrence of cardiotoxicity is remarkably rare when compared to other IRAEs; however, it is associated with a higher mortality rate.^7–10^ Consequently, in order to identify this uncommon consequence in patients treated with ICIs and initiate therapy promptly, a high level of suspicion is required.

Among cardiotoxicity, myocarditis is an increasingly recognized complication of ICI therapy, occurring in approximately up to 1.14% of individuals receiving either single or combination ICI therapy.^11^ It is noteworthy that most cases of myocarditis present within the first 1–2 months after initiation of ICI therapy, although clinicians should maintain a high index of suspicion even in patients on long-term ICI therapy.^5^ We present an unusual case of a 67-year-old male patient with a history of prostate cancer and renal cell carcinoma treated with ICIs (pembrolizumab and lenvatinib) presenting with myocarditis secondary to ICI therapy.

## Case presentation

A 67-year-old man, with a medical history of prostate cancer featuring a Gleason 3 + 4 = 7 adenocarcinoma and a prior recurrence of renal cell carcinoma managed with immunotherapy (pembrolizumab and lenvatinib), along with a history of supraventricular tachycardia, presented to the hemato-oncology clinic complaining of generalized fatigue progressing over 1 week. His heart rate (HR) was noted to be in the 50s (52 bpm), indicative of sinus bradycardia, which led to a reduction in his home verapamil dose from 360 mg BID to 360 mg daily.

Subsequently, he presented to the emergency department (ED) 3 days later with symptoms of fatigue, blurry vision, and bilateral lower-extremity weakness. He denied any complaints of chest pain, palpitations, syncope, fevers, and chills. He had no history of smoking or drug use and no familial history of heart disease. Upon examination, his blood pressure was 153/74 mmHg, HR was 59 bpm, and oxygen saturation was 99% on room air. Results of the physical examination were otherwise unremarkable.

Laboratory investigations, including complete blood count, comprehensive metabolic panel, and imaging studies such as chest X-ray and computed tomography of the spine revealed no significant abnormalities except for elevated aspartate aminotransferase at 668 U/L and alanine aminotransferase at 278 U/L. Creatine phosphokinase (CPK) was notably elevated at 13,000 U/L. Consultations were sought from hemato-oncology, rheumatology, ophthalmology, and neurology. Given the constellation of symptoms and findings, the patient was initiated on intravenous prednisone 80 mg/day for presumed immunotherapy-induced rhabdomyolysis/myositis. His presenting rhythm on his electrocardiogram (ECG) was sinus rhythm with first-degree atrioventricular (AV) block and Q-waves in septal leads.

Following admission, the patient experienced bradycardia with a HR dipping into the 30s (34 bpm), accompanied by diaphoresis and dizziness. An ECG revealed a sinus rhythm with first-degree AV block, non-specific intraventricular conduction delay, and elevated high-sensitivity troponin levels progressively increasing to 50,000 pg/mL. A transient episode of complete heart block with a 10-s pause before ventricular escape rhythm was recorded on telemetry overnight. A post-episode ECG demonstrated sinus rhythm with first-degree AV block, consistent with previous findings. The patient was transferred to the intensive care unit and initiated on low-dose dopamine, maintaining hemodynamic stability.

**Figure 1:**
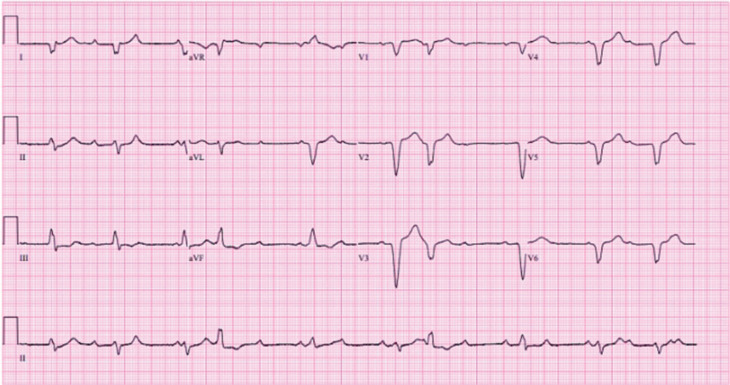
Electrocardiogram demonstrating heart rate before pacemaker placement.

**Figure 2:**
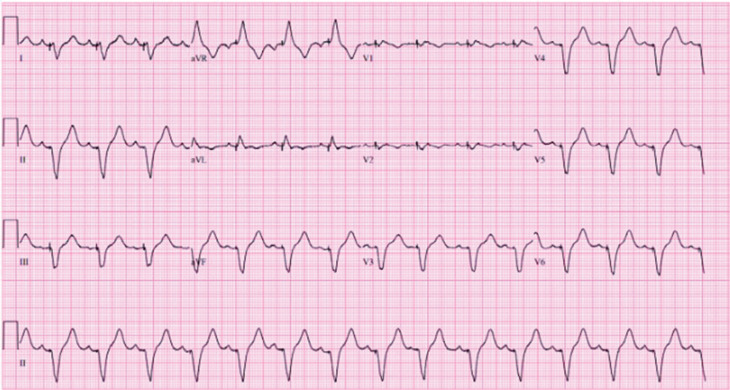
Electrocardiogram demonstrating heart rate after pacemaker placement.

Further evaluation with left heart catheterization revealed normal coronary arteries, ruling out ischemic etiologies. A temporary pacemaker was subsequently inserted, followed by permanent pacemaker placement. The ECG changes are shown in **Figures 1** and **2**. Additionally, the patient received treatment with prednisone and intravenous immunoglobulin. Within 2 days, the patient’s symptoms showed improvement. Ultimately, ICI (pembrolizumab)-induced myocarditis was identified as the underlying cause.

## Discussion

Use of ICIs has revolutionized cancer therapeutics. Although well-tolerated, there are several IRAEs that have been documented. Myocarditis is being increasingly recognized as a rare complication of ICIs, occurring in approximately 1.14% of individuals.^11^ The onset of symptoms has been variable among the documented cases. Mahmood et al. described a median time of onset of 34 days, where 81% of the patients developed ICI-associated myocarditis within 3 months of initiating the therapy.^9^ Altogether, the occurrence of myocarditis is common within the first 1–2 months after initiating ICI therapy, and diagnosis can be suspected even after one to two doses of immunotherapy.^10,12^

Combination ICI therapy, particularly the combination of anti–CTLA-4 and anti–PD-1 therapy, confers a 4.7-fold increased risk of developing myocarditis compared to single-agent therapy.^13^ Additionally, other agents, such as lenvatinib, a tyrosine kinase inhibitor, have also been associated with myocarditis and cardiomyopathy.^14^ The exact mechanism of ICI-related myocarditis is unclear; however, the proposed mechanisms include shared antigens between the tumor and myocardium or T-cell receptor targeting homologous muscle antigens.^8^ The mechanism is similar to viral myocarditis in which the myocardium is targeted by a process of molecular mimicry.^15^

ICI-associated myocarditis presents with a spectrum of clinical manifestations, including arrhythmias, heart failure (HF), or acute coronary syndrome (ACS) symptoms.^16^ Patients may also exhibit concomitant pericardial effusion with or without pericarditis. Notably, myocarditis has been reported more commonly in association with ICI-related myasthenia gravis and myositis, likely due to shared antigens between cardiac and skeletal muscles.^5^

Diagnosis of pembrolizumab-related myocarditis may pose a challenge. When suspected, lymphocytic myocarditis, dilated cardiomyopathy, and infections with cardiotropic viruses should be ruled out with a standard set of investigations.^17^ Endomyocardial biopsy remains the gold standard for diagnosing ICI-related myocarditis; however, it is limited due to its availability, especially in smaller community hospitals.^18^ Furthermore, it has low sensitivity and a high interobserver variability while interpreting the results of a biopsy sample.^19^ Therefore, clinicians often rely on clinical presentation, laboratory findings, imaging modalities, and ECG abnormalities for diagnosis.^18^ Addition of immunohistochemistry and polymerase chain reaction (PCR) analysis of the endomyocardial biopsy specimens may improve the diagnostic accuracy in patients with myocarditis.^19^ Our hospital did not have the facility for PCR and biopsy; hence, the diagnosis was limited due to the availability of resources.

In cases of ICI-related myocarditis with left ventricular systolic dysfunction, prompt initiation of HF treatment following current guidelines is imperative to improve patient outcomes.^20^ ECG abnormalities are often early manifestations of acute myocarditis and may carry significant clinical and prognostic implications. While most ECGs demonstrate non-specific and usually benign ST- and T-wave abnormalities, axis deviation, prolonged QRS duration, or premature ventricular contractions, serious arrhythmias or conduction disturbances such as AV conduction block are associated with a worse prognosis.^21^ Differential diagnosis for acquired AV block in young patients with suspected myocarditis includes various cardiac and non-cardiac etiologies, necessitating a comprehensive evaluation to ascertain the underlying cause.^22^ Furthermore, our patient may have had a synergistic effect on the cardiac autonomic system due to verapamil and lenvatinib on the development of arrhythmias.^23^ However, when the treatment for pembrolizumab-induced myocarditis was given using immunoglobulins, the symptoms improved. This suggested that the primary cause for arrhythmias was due to underlying inflamed myocardium secondary to pembrolizumab.

ICI-related myocarditis is managed with glucocorticosteroids, including both oral and intravenous routes.^24^ Patients who received high-dose glucocorticoids had a lower peak and discharge troponins; in addition, major adverse cardiovascular events were lower in patients who received high-dose compared to low-dose steroids in the study by Mahmood et al.^9^ The exact determining factors for a high or low dose of steroid is not clear; however, authors recommend a pulse dose of steroids at 1000 mg daily, followed by 1 mg/kg daily of either oral or intravenous steroids.^9^ The American Society of Clinical Oncology clinical practice guideline also suggests beginning at 1 mg/kg daily of either intravenous or oral steroid and tapering of at least 4–6 weeks in addition to holding off the ICIs.^24^ Thus, initiating steroids at a high dose, followed by a tapering with monitoring clinical response and troponin, can be used while treating ICI-related myocarditis. There have been few reported cases where a successful treatment was offered by using intravenous immunoglobulin, mycophenolate, and antithymocyte globulin.^25–27^ However, the effectiveness of these agents in managing ICI-related myocarditis remains unclear and can be used in those patients who have an inadequate response to glucocorticoids. In addition to immunosuppression, use of conventional cardiac therapy to manage acute decompensated HF and use of a temporary pacemaker for AV block may improve the clinical outcome in patients with myocarditis.^28,29^ Our patient was kept on a temporary pacemaker, following which he was placed on a permanent pacemaker after 2 days; this may be done in cases where the likelihood of spontaneous recovery is low, such as the case of pembrolizumab-induced myocarditis, where a partial improvement may reflect only partial recovery from myocarditis or stabilization of cardiac function without complete recovery from the underlying conduction.^30^ Hence, early implantation of a permanent pacemaker was justified in this situation.

## Conclusion

In conclusion, complete heart block secondary to ICI therapy poses a significant risk to patients with potentially fatal outcomes. Clinicians should maintain a high index of suspicion in patients presenting with myocarditis, elevated troponin levels, and prolonged QRS duration. Diagnostic evaluations encompassing elevated troponins, B-type natriuretic peptide, creatine kinase-MB, ECG, echocardiography, cardiac magnetic resonance, and endomyocardial biopsy are essential for accurate diagnosis. Management strategies involve prompt cessation of ICI therapy, along with early initiation of high-dose corticosteroids. Additional immunomodulatory agents such as mycophenolate and infliximab may be considered, while refractory cases may benefit from agents such as alemtuzumab, tocilizumab, or rituximab. Vigilant monitoring and timely intervention are imperative in optimizing patient outcomes in this challenging clinical scenario.

Overall, the diagnosis of acute myocarditis remains challenging due to the absence of pathognomonic clinical presentations. Clinicians must maintain a high level of suspicion, particularly in patients undergoing ICI therapy, and promptly initiate appropriate diagnostic and therapeutic interventions to improve outcomes in these high-risk individuals.
